# Retraction: Effect of varying thickness properties of the slow release fertilizer films on morphology, biodegradability, urea release, soil health, and plant growth

**DOI:** 10.1371/journal.pone.0328240

**Published:** 2025-07-16

**Authors:** The PLOS One Editors

The *PLOS One* Editors retract this article [1] due to concerns about peer review integrity and authorship. These concerns call into question the validity of the reported results. We regret that the issues were not identified prior to the article’s publication.

All authors did not agree with the retraction.
